# Enablers of Patient Knowledge Empowerment for Self-Management of Chronic Disease: An Integrative Review

**DOI:** 10.3390/ijerph18052247

**Published:** 2021-02-24

**Authors:** Vestina Vainauskienė, Rimgailė Vaitkienė

**Affiliations:** School of Economics and Business, Kaunas University of Technology, 44239 Kaunas, Lithuania; rimgaile.vaitkiene@ktu.lt

**Keywords:** patient knowledge empowerment, patient knowledge enablers, empowerment levels, self-management, chronic disease, health management, knowledge management, integrative review

## Abstract

The non-development of the concept of patient knowledge empowerment for disease self-management and the non-development of the theory of patient knowledge empowerment in patients with chronic diseases, cause methodological inconsistency of patient empowerment theory and does not provide a methodological basis to present patient knowledge empowerment preconditions. Therefore, the aim of the present integrative review was to synthesize and critically analyze the patient knowledge enablers distinguished in the public health management theory, the knowledge sharing enablers presented in the knowledge management theory and to integrate them by providing a comprehensive framework of patient knowledge enablers. To implement the purpose of the study, in answering the study question of what patient knowledge empowerments are and across which levels of patient knowledge empowerment they operate, an integrative review approach was applied as proposed by Cronin and George. A screening process resulted in a final sample of 78 papers published in open access, peer-review journals in the fields of public health management and knowledge management theories. Based on the results of the study, the Enablers of Patient Knowledge Empowerment for Self-Management of Chronic Disease Framework was created. It revealed that it is important to look at patient knowledge empowerment as a pathway across the empowerment levels through which both knowledge enablers identified in public health management theory and knowledge sharing enablers singled out in knowledge management theory operate. The integration of these two perspectives across patient empowerment levels uncovers a holistic framework for patient knowledge empowerment.

## 1. Introduction

The mission of the modern public health system is to improve the health of the population and reduce health inequalities through organized institutional and community effort. Chronic diseases are identified as a sustainability challenge for European health systems, as the growing scale of chronic diseases due to an aging population and increasing life expectancy requires increased financial investment and an effective response to patient needs and expectations. Meanwhile, research agrees that the paradigmatic shift in approach to chronic disease patients from disease-oriented to patient-oriented, as an active healthcare partner is important in this context [1]. The patient, as an active partner, could self-manage the disease, make rational, day-to-day decisions related to their health condition to ensure health behavior [2,3]. Health behavior in patients with chronic diseases reduces the financial burden on the healthcare sector [1,4], and active collaboration between patients and healthcare institutions facilitates the identification of patients’ needs and expectations.

An essential precondition for patient self-management of the disease is patient empowerment [3,5]. Patients are empowered to make independent disease management decisions and to be responsible, primarily when they have knowledge about their disease [3,5] and can use it purposefully in taking these decisions. However, the purposeful use of empowered patients’ knowledge needs specific preconditions called knowledge enablers.

There are several areas in the scientific debate on patient knowledge empowerment for self-management of disease. The concept of patient empowerment is still being developed; this highlights its heterogeneity and multidimensionality. In particular, the concept of patient empowerment is being developed in the contexts of different chronic diseases [2,4,5,6,7], which presupposes that the context of patient empowerment determines different means of patient empowerment to achieve long-term rational disease management solutions.

In the scientific discussion of concept development, a significant place is occupied by the isolation and analysis of patient empowerment results. Empowering patients for disease self-management also has a positive effect on the patient’s psychological state [3,4,8,9] interpersonal relationships [5,10,11,12,13] and ultimately manifests itself through health behavior. To achieve the results of patient empowerment in the healthcare system discussed above, it is important to create preconditions for patient empowerment for disease self-management.

Research on patient empowerment to date from a public health management perspective has focused on individual patient enablers without substantiating them as potential patient knowledge enablers: digital health technologies [14,15], availability of reliable information, digital health communities, and others. Knowledge management theory discusses knowledge sharing extensively and in detail, distinguishing preconditions that ensure knowledge sharing: supportive organizational culture [16], organizational member motivation [17], mutual trust [18], less centralized organizational culture [19], etc. In this context, Ippolito et al.’s (2020) systematic literature study and Scharf’s (2014) Knowledge Management Model of Patient Learning stand out [13,20].

Ippolito et al. (2020) distinguished common, but not knowledge-related, groups of enablers of patients with chronic diseases: patient learning and knowledge, social support and counseling, developing and maintaining relationships with patient ecosystem stakeholders, and patient-centered healthcare models [13]. Scharf’s (2014) knowledge management model for patient learning identifies and integrates interorganizational and environmental enablers and critical knowledge management processes based on and developed by classic knowledge management theory: active and passive knowledge discovery, explicit and implicit knowledge sharing, and knowledge creation [20]. However, it remains unclear whether the identified knowledge enablers are relevant to the knowledge empowerment of patients with chronic diseases.

It should be noted that one of the latest directions in research is the development of a conceptual model of the Learning Health System [21,22,23,24]. A key value proposition of the learning health system is patient-centeredness through rational and health-friendly decision-making in the lifelong learning process involving patients themselves and key stakeholders of their ecosystem [21]. Despite the fact that the continuous knowledge creation and its effective empowerment are reflected in the whole concept of the learning health system, neither the concept of patient knowledge nor that of patient knowledge empowerment is developed in scientific works.

Knowledge is multidimensional in its nature because, from a classical theory of knowledge management, knowledge includes explicit, tacit, and dormant knowledge [25]. In order to create knowledge, a continuous cyclical process takes place, integrating various knowledge management activities that stimulate the processes of knowledge acquisition, conversion, and use and in knowledge management theory these activities are widely analyzed and developed as knowledge enablers [26,27].

Thus, the field of research in public health management theory develops patient empowerment more from a patient perspective, while the field of knowledge management theory presents enablers who stimulate knowledge management processes from an organizational perspective.

To date, patient empowerment research faces problems of fragmented approach and insufficient conceptualization, as the lack of a framework for patient empowerment in patients with chronic diseases causes methodological inconsistencies in patient empowerment theory and does not provide a methodological basis for presenting knowledge empowerment preconditions. However, the integration of public health management and knowledge management theories would provide a comprehensive framework for patient knowledge enablers.

The objective of the present integrative review was to synthesize and critically analyze the patient knowledge enablers presented in the publications on public health management, the knowledge enablers presented in the knowledge management theory and to integrate them by presenting the enablers of patient knowledge empowerment for self-management of chronic disease framework. Thus, the integrated approach of the two different fields applied in this study significantly contributes to the consistent development of the topic of patient empowerment, as the results obtained capture the depth and breadth of the patient empowerment theory and contribute to a new understanding of the phenomenon of concern.

## 2. Materials and Methods

In order to fulfill the goal of the integrative review, primarily, the approaches of patient knowledge enablers are selected and substantiated, and the methodological choices of the integrative review are substantiated.

### 2.1. Patient Knowledge Enablers: Theoretical Background

In order to theoretically substantiate knowledge enablers for patients with chronic diseases, first, it is important to reveal the aspects of empowerment in its broad sense and the levels across which patient empowerment develops, then to understand the mechanism by which the patient, across the levels of empowerment, acquires the power to purposefully use their knowledge in their day-to-day decisions to achieve health behavior. Finally, to identify why, in addition to patient knowledge enablers emerging from the field of public health theory research, the knowledge enabler, widely analyzed in the field of knowledge management theory research, driven by the interaction of members of the organization knowledge sharing is particularly important.

#### 2.1.1. Patient Empowerment and Its Levels

In the theory of empowerment, one of the main keywords is power, and its concept is revealed through the ability to get what is needed; to influence how others think, feel, behave, and what they believe in; to allocate resources in social systems such as family, organization, community, and society [28]. Gutiérrez et al., (1995), summarizing the insights of a number of authors, present essential aspects of the concept of empowerment [28]:

- Empowerment is both theory and practice that deals with aspects of power, powerlessness, and oppression and how they contribute to the problems of individuals, families, or communities and affect helping relationships.
- Empowerment aims to increase personal, interpersonal, or political power in such a way that individuals, families, or communities can take action to improve their situation.
- Empowerment is a process that takes place at the individual, interpersonal, and/or community levels and includes subprocesses such as developing group awareness, reducing self-blame, accepting personal responsibility for change, and improving self-efficacy.
- Empowerment occurs through intervention methods, basing help relationships on cooperation, trust and shared power, awareness raising, individual involvement in the process of change, training in special skills, and mobilization of resources.

Thus, effective empowerment practices are not fighting or adapting, but increasing real power so that individuals are able to protect themselves from the problem or change it. As Peterson (2014) emphasizes, empowerment is an active, participatory process through which individuals gain greater control over their lives, acquire rights, and reduce marginalization [29].

Scientific discussions reveal that patients are empowered when they have the knowledge, skills, attitudes, and a certain level of self-awareness to influence their behavior and to cooperate effectively with stakeholders to achieve optimal wellbeing [30]. The context of chronic diseases means that the specific expertise of patients is formed simply because they are forced to live every day with the symptoms and consequences of their disease and to communicate periodically with healthcare professionals (passive involvement, the knowledge is rather tacit here). According to Bate and Robert (2006), patient engagement in their disease management evolves: first, patients take on the role of those who complain, then provide information about their conditions, listen and respond, counsel, and advise until they finally fully participate and become involved in taking chronic disease management decisions [31].

Empowering patients for independent disease management also has a positive effect on the patient’s psychological state through patient self-confidence [4,8,9], positive self-perception [9], and self-esteem [3]. Research also highlights the results of patient empowerment via the social dimension, as empowering the patient to act independently has a positive effect on his interpersonal relationships with relatives, healthcare professionals [5,10,11,12,13], and communities of patients with the same disease [32]. Finally, the empowerment of a patient with chronic illness manifests itself in their behavior through conscious internal control of behavior self-efficacy, which allows them to make rational decisions for self-management of chronic illness and is a key precondition for health behaviors [9,33]. Wagstaff (2006) distinguishes three models of patient involvement in decision-making [34]. In the traditional paternalistic model, decisions related to patient‘s health are made by the healthcare professional with minimal information to the patient. In the shared decision model, the patient participates in the decision-making process by expressing their preferences among the possible solutions. In the informative model, the healthcare professional provides all the necessary information for the patient to make a choice. In this context, it is important to emphasize that the pursuit of patient knowledge is dissociated from the involvement in the decision-making process in a patient-centered approach, the pursuit of patient consultation does not imply a shared decision-making model.

As patients are actively involved in decision-making related to their health, there is a need to identify existing knowledge, acquire new knowledge, develop it, share it with stakeholders in the ecosystem, and use it to make effective disease management decisions.

Patient empowerment can take two forms: at the individual level, when a patient identifies themselves with a chronic illness, has the necessary knowledge and control, and can make decisions; at the community level, where patients can empower other patients in the community by disseminating their knowledge, experience, etc. [35]. However, the perception of the community varies depending on the level of empowerment.

Rissel (1994), like many other authors, takes the view that maintaining community health is inseparable from community empowerment. Above all, however, empowerment begins and develops at the individual level, is characterized by participation in informal patient communities, raising awareness, and ultimately concrete social action in community organizations [36]. Rissel (1994) presents Torre’s (1986) view that community empowerment develops through three main components, and that without at least one of them, community empowerment is not possible [36]:

- A microcomponent covering such intrapersonal aspects as patient self-esteem and self-efficacy;
- Mediating structures characterized by mechanisms specific to groups of individuals and active participation of group members in sharing knowledge and growing their critical consciousness.
- A macrocomponent that encompasses social and political activities as mediating structures become community organizations capable of changing or creating new social conditions.

The isolated levels of patient empowerment presuppose that the microcomponent of patient empowerment reflects the psychological empowerment of patients, which, when the patient identifies themselves with a chronic disease, has the necessary knowledge and control and can make informed decisions and for which participation in collective political action is unnecessary. Meanwhile, the empowerment of community, first through mediating structures, and later through community organizations, is possible with raised level of psychological empowerment among individual members of the community.

In the context of modern organizational theory, an organization is treated as a system of interrelated components such as individuals, their formal and informal groups, and their patterns of behavior arising from the needs of the organization; perception of personal role within the organization and physical environment in which individuals act to achieve organizational goals. All these components are combined by linking processes, which are aimed at the most effective interaction of the mentioned components to achieve the goals of the organization: communication, balancing between the components for balanced operation and decision analysis [37]. 

Dizon (2012) presents a definition of community organization by Kramer and Specht (1975): “Various methods of intervention, whereby a professional change agent helps a community action system composed of individuals, groups, or organizations to engage in planned collective action in order to deal with social problems within a democratic system of values. It is concerned with programs aimed at social change with primary reference to environmental conditions and social institutions” [38]. In the context of patient empowerment, community organizations are based on an active participatory decision-making model to achieve community-important goals in the perspective of health improvement. Communities devise various programs to implement health-related goals by concentrating on their strengths and using collective effort. Based on this perception, it can be argued that community organization is a system characterized by the components of the organization singled out in the context of modern organizational theory and the processes that connect them, aimed at achieving a common goal. 

Thus, the levels of patient empowerment discussed above by Rissel (1994) can be identified with the distinct components isolated by modern organizational theory (first, there is an individual, then under specific preconditions informal (usually) patient groups form until a formal organization is finally set up) and therefore are ensured by the same linking processes [36].

Patient empowerment takes place across levels of empowerment, and the result of patient empowerment is primarily the psychological empowerment of individuals and then that of community by creating organizations involving stakeholders, which operate through interaction to achieve health-related goals.

#### 2.1.2. Patient Knowledge Formation for Patient Empowerment

Paulo Freire, who coined the concept of patient empowerment, has linked patient empowerment primarily to a process where educational intervention focuses on shaping patients’ ability to think critically and act autonomously [4]. Thus, from a patient perspective, their empowerment can be interpreted as a process of empowering the patient to make independent decisions about their illness in routine situations [10]. Small et al. (2013) look more globally, arguing that the empowerment process is the patient’s participation in healthcare [2]. Through the empowerment process, the patient, from the passive recipient of information becomes an active healthcare partner, able to select from the abundance of information and make the most appropriate decisions for the course and condition of his disease [5]. In other words, it is in the process of empowering the patient that the patient’s knowledge is created and used by the patient to implement effective decisions related to his or her health. There are three main implementation-oriented approaches in research [39]:

- Promoting Action on Research Implementation in Health Services (PARIHS);
- Consolidated Framework for Implementation Research (CFIR);
- Knowledge to Action Framework (KTA).

All of these approaches concentrate on and interpret the process of implementation in their own way, but only the core of the KTA approach is the knowledge with which specific actions can be effectively implemented in practice.

The KTA approach provides conceptual guidance on how to integrate the stages of knowledge creation with knowledge implementation [40]. In the process of funnel-shaped knowledge creation, knowledge is as if distilled (knowledge inquiry, knowledge synthesis, and knowledge tools/products) until clinical practice recommendations or patient decision aids are created. The action cycle consists of seven stages and provides for the use of distilled knowledge: (1) identify the problem and determine the know/do gap; identify, review, and select knowledge; (2) adapt knowledge to the local context; (3) assess barriers/facilitators to knowledge use; (4) select, tailor, and implement interventions; (5) monitor knowledge use; (6) evaluate outcomes; and (7) sustain knowledge use.

The stages of the action cycle can occur sequentially or overlap, and the cycle of knowledge formation can influence the action cycle at any stage. The elements of the action cycle focus on deliberately bringing about change in healthcare systems and groups [32,33].

Based on the discussed approach and the above insights on empowerment in general and patient empowerment, it can be stated that patients’ knowledge is formed in a process that includes the search for knowledge about their disease, its synthesis and the use of specific knowledge. Meanwhile, the action cycle is a process of knowledge empowerment, in which the patient, through the levels of empowerment, acquires the power to purposefully use their knowledge in everyday decisions to achieve health behavior.

#### 2.1.3. Patient Knowledge Enablers: The Importance of Integrating Public Health Management and Knowledge Management Theories

Knowledge enablers are generally defined as processes, inputs or sources facilitating the manipulation of knowledge. The concept of the health system is inseparable from the concept of patient empowerment. The health system includes all organizations, people, resources, and actions whose primary goal is to promote, restore, and maintain health at the individual or population levels [41]. Both the structures and processes of the health system (as supply factors) and patient choices (as demand factors) determine the progress of patient health. The patient’s movement through the health system is identified with a pathway that describes the patient’s progress through the health system to avoid side effects and complications in the context of chronic disease. According to Brathwaite et al. (2020), the patient uses a variety of enablers on their path through the health system to achieve health behavior [41]. From the perspective of conceptualizing patient knowledge, patient knowledge enablers create context and provide stimulating preconditions for patients to use knowledge through empowerment levels in the knowledge to action framework action cycle to make a specific decision. In addition to these enablers, it is particularly important in the context of patient empowerment that as a patient travels through levels of empowerment, the use of their knowledge is stimulated by purposeful interactions with other patients with chronic illness.

Classical knowledge management theory assumes that the creation of knowledge involves a continuous cyclical process that integrates a variety of knowledge management activities and solutions focused on the use and continuous updating of knowledge. As already mentioned, in order to enable patients to self-manage chronic disease in daily life, it is important not only to develop the necessary knowledge but also to develop cognitive abilities to use knowledge for rational routine decision-making and constantly update it. The essence of knowledge creation is to generate new solutions, form, develop, and use competencies using knowledge management tools [25]. Knowledge management theory provides management solutions that can be adapted to empower patients’ knowledge to create preconditions for independent disease management.

Enabling factors for knowledge management are vital infrastructure for the effectiveness of knowledge management activities. According to Kale and Karaman (2011), these are organizational mechanisms that stimulate the creation and development of knowledge in an organization and facilitate the creation, conversion, use, and protection of knowledge [27]. In knowledge management theory, knowledge empowerment has been developed through different knowledge management processes in an organization, which have been singled out by Gold et al. (2001) and whose enablers are extensively studied in knowledge management theory [42]:

- The process of knowledge acquisition, which includes knowledge creation, search, and collaboration activities [43,44];
- The process of knowledge conversion involving activities of knowledge organization, storage, integration, and coordination [45,46];
- The process of knowledge use/application, including retrieving and knowledge sharing [20,47];
- The knowledge protection process that manifests itself through knowledge storage activities [42].

An organization’s knowledge management capabilities, helpful to adapt to an uncertain external environment, are shown in how effectively an organization can acquire, retain, and distribute knowledge. In other words, knowledge management skills are related to the development of specific enablers that create the context for knowledge management activities knowledge exploration and knowledge exploitation. Knowledge sharing is at the heart of the classic spiral SECI model, which encompasses the following knowledge creation processes that take place in the interaction of members of an organization: in the process of socialization through shared experience without the use of language tacit knowledge is created; externalization is directed at the conversion of tacit knowledge into explicit knowledge; the process of combination involves the sharing and coordination of the explicit knowledge, thus creating new knowledge; the process of internalization is the conversion of explicit knowledge into tacit knowledge and reflects the essence of learning [44]. Thus, in knowledge management theory, the preconditions of knowledge sharing in an organization are widely discussed because the authors unanimously agree that knowledge sharing is a critical organizational capability integrating other knowledge management practices and processes that result in organizational sustainability [17,26,48,49,50,51]. Therefore, this article assumes that in addition to patient knowledge enablers emerging from the field of public health theory research, the knowledge enabler, widely analyzed in the field of knowledge management theory research, is especially important, driven by the interaction of the organization’s members knowledge sharing.

Keeping this view in mind, the present review paper mainly focused on two theories public health management and knowledge management and on the interdisciplinary approach provided by these theories. The synergy of these theories would lead to a fulfilled framework of patient knowledge enablers, answering the research question What are patient knowledge enablers and through which levels of patient knowledge empowerment do they operate?

### 2.2. Methodology of Literature Review

To answer the research question, Cronin and George’s (2020) integrative review approach was used as one of the most recent one, generalizing many previous integrative review approaches, and, according to the authors of this article, easily adaptable to synthesize scientific literature from different scientific theories to capture a phenomenon evolving in the context of different perspectives [52]. The authors of the article also followed the stages of integrative review as suggested by Cronin and George (2020): (1) choice of synthesis vehicle, which can be adjudication and redirection; (2) literature review; and (3) thematic synthesis [52]. The following describes all integrative review decisions that were made in gathering the data.

#### 2.2.1. Choice of Synthesis Vehicle

According to the above-mentioned authors, adjudication is the most adequate to use not for meta-analyzes but for systemic analyzes, where the aim is to reveal causal relationships based on objective quantitative constructs and systematization standards. The essence of this research question lies in the desire to unite multiple communities of practice. Therefore, to answer the research question addressed in this article, redirection is a more appropriate strategy because, according to the authors mentioned above, it is applied when seeking to find a new insight about a topic through a juxtaposition of several studies, including “disciplined imagination to develop new kinds of ideas that are necessarily speculative out of current domain knowledge, and it foregrounds aspects of the domain in need of more frontline empirical work ” [52]. Thus, the application of redirection vehicle provides space to raise new questions and thus steer future research in unexpected directions.

#### 2.2.2. Literature Review

According to Cronin and George (2020), in the literature review phase, it is important to ensure the completeness and balance criteria of the selected literature [52]. In the context of an integrative review, a complete review is important, i.e., it is important for researchers to analyze all literature that met the search criteria, including previously unexplored and unknown concepts and methodologies. Having ensured the completeness criterion, both a quantitative and a qualitative balance between different paradigms is sought after, as each of the paradigms is based on a different social–scientific reality.

To ensure both criteria of the literature review, the selection of research papers was performed in two databases of research covering different scientific theories. The ScienceDirect database includes scientific journals from a variety of disciplines, including Health Sciences (public health management); The Emerald Management database includes full-text journals in the field of social sciences (management science).

Both of the authors of the present study independently carried out a search in these two databases. The search string used in the present study was the following:

- ScienceDirect database search string: “patient empowerment” AND “knowledge” AND “enablers”.
- Emerald Management database search string: “knowledge management enablers” AND “empowerment”.

It should be noted that keywords related to chronic diseases were not included in the search string because of the tradition in public health management work that the concept of patient empowerment is inseparable from the context of chronic diseases.

The search strings were modified slightly in several cases to function properly on some search platforms. When searching for research papers in the ScienceDirect database, it was chosen to include scientific articles from 2008 to September 2020. The year 2008 was chosen as a starting point because it was noticed that it was from that period that the topic of patient empowerment began to appear in this database. Meanwhile, knowledge sharing, as one of the knowledge management activities, has a long research tradition, so the starting point of the year in the Emerald Management database was the emergence of the database, and research papers were sought until September 2020.

**Figure 1:**
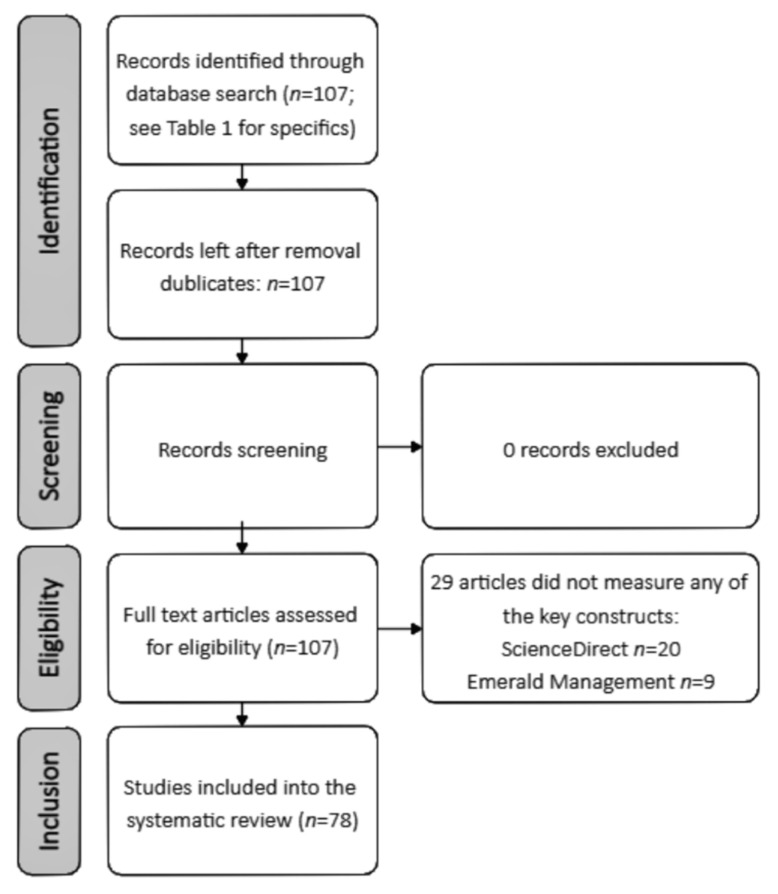
Article screening and inclusion procedure.

It should be mentioned that since the research papers were searched for in two databases on different fields of science, no duplicate articles were found. After making sure that there were no duplicate research papers, the authors of the article independently screened the titles, abstracts, and keywords of the selected research papers and if no connection was found with patient empowerment, knowledge, enablers (in case of search a.), and with knowledge management enablers, empowerment (in the case of search b.), they were eliminated from further analysis. When there was no clarity as to whether the article could be included, it was read in full. Careful reading was done of the articles that were chosen as eligible, and those not relevant were not included in further analysis. Figure 1 presents a step-by-step representation of our screening process.

Studies were included in the analysis if they met the eligibility criteria:

- As the aim of the study was to find a new approach that integrates two different research traditions, to reveal the holistic approach and ensure the completeness criterion, it was important to find a wide range of knowledge enablers; therefore, the research that conceptually and/or empirically develops the above keywords from various perspectives were considered suitable;
- The Emerald Management database does not detail search results and only provides research papers. In order to ensure a balance between the two disciplines when analyzing only scientific publications, the types of publications such as conference abstracts, mini reviews, short communications, and Encyclopedia were not included in the data analysis from the results obtained in the ScienceDirect database. From both databases, the research included in the integrative review met the peer-review and open access criteria. The application of the peer review filter in the search of both databases resulted in the entry of valid research papers into the integrative review.

The total number of articles that matched the search string from each database is presented in Table 1. A similar number of publications were selected in both databases, which suggests that the applied search string combinations and eligibility criteria allowed one to balance the literature review results both quantitatively (article type) and qualitatively (peer review).

#### 2.2.3. Thematic Synthesis

According to Cronin and George (2020), at the thematic synthesis stage, broader themes from the different perspectives were abstracted and integrated to support the goals of the review (adjudication or redirection) [52]. By abstracting themes in different perspectives, it is important for the researcher to discover ones that connect different research traditions, because only in this way they can be combined into a single whole, i.e., integrated. When integrating themes from different research traditions, it is important to highlight the connections between the themes, iterating as much as necessary to find a “maximally elegant framework” that highlights relationships among themes more than within them.

The computer-assisted qualitative data analysis software MAXQDA 2020 (VERBI GmbH, Berlin, Germany) was used to analytically summarize the qualitative data by distinguishing the themes relevant to this study and the relationships that integrate them. The developers of this program identify its numerous benefits and especially wide range of useful functions: qualitative data interrogation, reflection, integrity, and exploration [53]).

Given that the research object has not been studied, an inductive coding approach has been applied, where research themes arise from qualitative data by constantly reading and thinking about it in an iterative process of qualitative data analysis, which, according to Seidel (1998) has characteristics such as repeatability and progression, recursivity, and holography [54].

## 3. Results

The thematic synthesis of the selected research papers took place in the following sequence: first, the research papers on public health management selected in the Science Direct database and the research papers on knowledge management selected in the Emerald Management database were coded separately. With the help of computer-assisted qualitative data analysis software, in identifying the relationships between patient knowledge enablers and patient empowerment levels, the themes from the research of both theories integrated through patient empowerment levels were identified.

### 3.1. Overview of Identified Themes

From the synthesis of reviewed articles, the following important themes were identified as shown in Table 2 and Table 3. As can be seen in the tables, the literature on both theories is dominated by specific subthemes.

The analyzed public health management research papers widely discuss patients’ self-care through empowering forms of healthcare. This subtheme is closely related to healthcare technologies, which are a particularly important part of modern health care and a dominant and transforming trend in the health care system of the future.

In the analyzed knowledge management research papers, the subtheme of organizational culture supporting knowledge sharing was especially distinct. Unsurprisingly, the authors pay a lot of attention to organizational culture, as it is identified in the management paradigm as one of the most important factors in ensuring organizational sustainability in the context of transformational change caused by industry 4.0.

In the qualitative analysis of the selected research papers, the levels of patient empowerment were coded as the subthemes, and the literature of both disciplines were combined through the above-mentioned subthemes. As can be seen in Table 4, most citations were coded to justify empowerment through mediating structures and empowerment at the macrolevel. This suggests that the research analyzed in both theories focuses more on formal and/or informal groups of individuals as components of the organizational system.

### 3.2. Patient Knowledge Enablers: A Public Health Management Perspective

#### 3.2.1. Individual Patient Knowledge Enablers

Patient autonomy is a subtheme based on the highest number of citations in the theme of patient individual knowledge enablers. In the context of patient empowerment, it is important to emphasize that patient autonomy in making health-related decisions does not mean that the patient has complete independence and the healthcare professional or other stakeholders are not involved in decision-making [55,56]. Meis et al. (2014) explain this by the term “autonomously dependent”, arguing that the positive effect of patient empowerment also occurs when the patient knows that in situations where a risk-free health-related decision is needed, their knowledge ensures optimal decision-making [56]. However, the patient is also aware that in a more risky situation, they are not left alone and can turn to a healthcare professional to apply a shared decision-making model and reduce the risk to the patient. Patient autonomy allows them to feel confident in their knowledge, motivated to obtain even more knowledge from a healthcare professional [57], and therefore encourages involvement in formal and informal patient organizations that ensure the uptake of digital health-related information through knowledge sharing [57] and the transition from the level of microempowerment to empowerment through mediating structures [58].

According to Meis et al. (2014), involving the patient in the search for and making a health-related decision essentially means that the patient will not only face the final, likely a good outcome of the decision-making, but will also experience barriers in the decision-making process, and will not avoid unforeseen constraints and thus will learn to look for alternatives and tackle barriers, i.e., discover knowledge and share it with those involved in the decision-making process [56]. In the context of empowerment theory, equal participation of patients in the decision-making process is like a means of encouragement and self-efficacy.

In the research papers analyzed, the subtheme of the patient‘s partner‘s involvement as an enabler of knowledge sharing was singled out. With certain chronic diseases, day-to-day healthcare decisions are inseparable not only from the patient but also from those close to them, who often not only share information about the patient’s physical environment and daily conditions to maintain the effectiveness of the treatment, strengthen patient and healthcare professional‘s therapeutic relationships, and reassure patients [14].

The effective participation of the patient in shared decision-making is also ensured by the available clear information on the nature of the disease and the treatment offered, preconditions for medical success, risks and side effects, reasonable treatment alternatives, their risks and side effects and possible consequences if not treated with the best alternatives. Having clear and structured information enables the patient to apply the available knowledge in making the best treatment decision and behaving healthily in daily activities. According to Menon and George (2018), patient empowerment increases the level of patient self-esteem that can be treated as an enabler of patient knowledge because a patient’s awareness that he has sufficient knowledge and can apply it determines their confidence in the treatment, which directly affects the effectiveness of the treatment [59].

#### 3.2.2. Health Technologies

The importance attributed to health technologies in the research analyzed can be explained by the digital transformation in many areas and the consequent digitization of medical and public health and the empowerment of patients through digitization.

Digital self-care through empowering forms of healthcare: The digitalization of medicine and healthcare allows for the development and implementation of new forms of healthcare (e.g., Mobile Health Tools, Clinical Decision Support Systems, Health Information Systems, and Do It Yourself Technologies) in which patients themselves are actively involved and which require active patient participation in both the development and application of the tools in day-to-day healthcare. In other words, the digitalization of healthcare is shaping the trend of digital self-care, where patients have the opportunity to play an active role in the day-to-day management of chronic disease through interactions with digital forms of healthcare [60]. The use of digital devices increases patients’ ability to follow a medical regime and recommended lifestyle through the integration of these forms into the daily activities of the patient [57] while supporting the functions of reminder, feedback, knowledge creation, and self-care [14]. As a result, patients’ routine decision-making becomes smoother, building patients’ self-confidence [14,15,59,60,61,62,63] and highly dynamic [63,64,65]. 

Enabling forms of healthcare are closely linked to patients ’access to personal health data. Health technology means that medical information about a patient becomes available not only to healthcare professionals but also to patients. The availability of personal information supports the patient’s empowerment to manage their health [66] and the interactions between health professionals in different fields [64,66]. Thus, the emergence of the digital revolution in medicine and new self-care technologies are narrowing the boundaries between expert and patient practice [67].

Availability of reliable information: There is a clear consensus in the analyzed research that certified and high-quality digital health technologies provide patients with reliable information about their disease [14,59,64,65,68]. The availability of reliable information leads to the consistent development of knowledge by empowered patients and is therefore more insightful about their health, leading to more informed decisions, early diagnosis, and faster recovery.

Creating e-communities. According to Menon and George (2018), one of the most essential tools to effectively empower a patient for chronic disease management is the ability to access and participate in virtual patient communities [59]. The accessibility of these communities allows for communication with patients with the same disease and health professionals when all three essential processes of knowledge management take place: knowledge acquisition, knowledge conversion, and knowledge use [60,67,68].

Health technologies can be seen as enablers of patient knowledge because their use to self-manage chronic disease in the daily lives of patients not only facilitates the process of building patient knowledge, but also enables the patient to actually act using their knowledge in everyday decisions for health behavior.

#### 3.2.3. Patient-Centered Healthcare Model

The paradigm shift from disease-orientation to patient-orientation is identified in research as a patient-centeredness model, which is defined as healthcare that respects the individual patient and responds to the individual patient’s preferences, needs, and values and ensures that patient values guide all clinical decisions [69]. In essence, the goal of this model is to engage the patient in their chronic disease treatment and day-to-day management decisions in the way that is most acceptable to them.

The Picker Institute singled out assumptions that together define the essence of patient-centered care, and one of these dimensions focuses on the importance of timely information and patient education. The research emphasizes that it is particularly important for healthcare professionals to provide more than enough knowledge to patients with chronic diseases that will help them understand the treatment scenario and how much effort is needed to treat the disease [59]. Van der Heide et al. (2018) emphasize that in the patient-centered care model, patient resources are particularly important: patients with strong self- efficacy, high levels of health literacy, and a broad social network are more able to manage their health conditions and care situations [69].

The active involvement of the patient in their healthcare can be seen as an enabler of patient knowledge, because in such a healthcare model, patient knowledge is managed on a continuum basis: patient knowledge that enables the patient to act effectively in their everyday life by utilizing the knowledge when it is necessary is continuously created (i.e., facilitates manipulation of knowledge).

#### 3.2.4. Disease Management Programs

Disease management programs are structured treatment plans designed to help chronically ill patients manage their chronic disease and maintain and improve quality of life. Pimouguet et al. (2011) define disease management programs as an ongoing process and proactive patient monitoring involving at least two of the following components: patient education (nutrition and physical activity recommendations and self-monitoring and knowledge related to disease and medication), coaching (providing tools to the patient to overcome psychological and social barriers to independence or adherence to treatment), adjustment of treatment (disease manager can start a new treatment or modify an existing one without/with the prior consent of the primary care physician), monitoring (health professional receives medical data from the patient), and care coordination (the health professional reminds the patient about upcoming visits or important aspects of their healthcare and informs the physician of complications, treatment adjustments, or therapeutic recommendations) [35,70,71],

Executing a disease management program essentially means that a patient in constant interaction with a healthcare professional constantly acquires knowledge, converts it, uses it, and evaluates it. The evolving ability of patients to evaluate means that they are not only able to evaluate knowledge from a variety of perspectives, but, above all, to apply and analyze it in everyday decisions disease-related. Thus, through the disease management program, the patient, as an enabler of knowledge, becomes an active healthcare partner, able to select from the abundance of information and make the most appropriate decisions for the course and condition of their disease.

#### 3.2.5. Learning Health System

Despite its broad coverage, the healthcare system has emerged in the research papers analyzed as a context that facilitates patient manipulation of available knowledge. According to the analysis of the research, the mission of the healthcare system in the context of chronic diseases is to create and maintain access to evidence-based resources for health care professionals and patients, develop the necessary knowledge and skills, reduce emotional stress, maintain combat and self-efficacy skills, and the application of health technology tools, thereby creating and maintaining patients’ quality of life [15,59,72,73]. This insight presupposes that, without naming it specifically, the authors are talking about the learning health system. The core value of a learning health system is patient orientation through rational and health-friendly decision-making in lifelong learning, involving patients themselves and key stakeholders in their ecosystem (e.g., family, patient communities with the same disease, treating physicians, etc.), thus ensuring continuous improvement of and innovation in healthcare. The learning health system uses the latest technological advances to achieve these goals to ensure dynamic collection and implementation of medical evidence (confirmation), rapid learning based on daily patient care data and thus leading to higher quality, safety, and innovation in healthcare. Thus, the continuous development of knowledge and its effective use in this way empowering patients is reflected in the whole concept of the learning health system [21].

Patient activation by enabling knowledge for day-to-day decisions related to chronic disease management takes place in the context of the learning health system dominated by the patient-centered healthcare model where the patient is an active partner whose preferences, needs and values are taken into account; forms of healthcare based on digitized health technologies are used and chronic disease management programs are in place, which help to respond to the patient proactively and ensure the processes of patient knowledge acquisition, conversion, and use.

Summarizing the themes reflecting the enablers of patient knowledge from the perspective of public health management, it can be stated that, in principle, the empowerment of patients takes place through the process of knowledge sharing, applying technological solutions. The process of harnessing knowledge involves the rapid discovery and sharing of existing knowledge. Knowledge discovery activities, as its use activities, are less relevant in the context of knowledge empowerment for patients with chronic diseases than knowledge sharing, since patients can quickly discover the required knowledge through health technology solutions. Health technologies create mechanisms and knowledge repositories to ensure that the knowledge needed to make a decision is accessed quickly. Isolated chronic patient knowledge enablers act as prerequisites to facilitate patient knowledge empowerment through direct (e.g., disease management programs) and indirect (e.g., digitized forms of healthcare) interaction between patients, healthcare professionals, and patient e-communities through knowledge sharing. The process of knowledge sharing is the transfer of knowledge to another individual as needed for proper decision-making [51]. It is a two-way process patients communicate through health technology tools with healthcare professionals and other patients, provide knowledge about their disease, and receive the response they need, thus expressing knowledge and combining it to make a specific health-related decision [18,74].

### 3.3. Patient Knowledge Enablers: A Knowledge Management Perspective

#### 3.3.1. Technological Knowledge Sharing Enablers

In order to share knowledge, it must first be codified, i.e., tacit knowledge is translated into explicit knowledge and this, in the context of digitization trends, is made fast using such technologies [51,75,76] Qandah et al., 2020), as Web 2.0 [17,51,74] and knowledge sharing systems [16,26,50]. In the analyzed management literature, Web 2.0 as a knowledge sharing tool is related to social networking, blogs within the organization, virtual communities of practice, expert profile systems, and the intranet [53]. According to the classical theory of knowledge management based on Nonaka and Takeuchi (1995), tacit knowledge, which is usually developed in organizations over many years, is extremely difficult to codify and therefore sharing it at horizontal and vertical organizational levels [77] requires a shared social process and informal face-to-face interaction between members of the organization, which is activated by the members of the organization acting in a specific environment created for knowledge sharing. If such an environment is not created, knowledge management in an organization simply cannot exist [50].

The analyzed research papers found a connection between the specific environment necessary for knowledge sharing and the use of Web 2.0 tools. According to Arif et al. (2015), social networks create an informal knowledge-sharing environment that encourages interpersonal relationships and collaboration among members of an organization that does not restrict communication due to differing social norms, cultural values, and interests [17]. Malik and Kaval (2018) emphasize that information technology support and application intensity fundamentally and drastically change knowledge-sharing behavior [75].

Thus, the main purpose of the technology-based tools discussed is not simply to access data and information, but to support the interaction between members of the organization and, as a result, the transformation of individual tacit knowledge into organizationally explicit knowledge and vice versa.

#### 3.3.2. Systematic Knowledge Sharing Enablers

The study of research has revealed that knowledge sharing in an organization does not happen by chance specific preconditions must be created in the organization to ensure the process of knowledge use through knowledge sharing.

Supportive organizational culture: Organizational culture is a critical precondition for securing knowledge management activities, but one of the biggest challenges is to develop a type of organizational culture that promotes knowledge management activities [49]. There is a relationship between the culture of the supportive organizational culture and the attitudes of the members of the organization towards knowledge sharing within the organization [51,78]. It should be noted that in this theme there are distinctive characteristics of the supportive organizational culture that have a positive effect on knowledge sharing practices: openness to change, promotion of innovation, trust, teamwork, high moral standards, information flows, involvement, supervision, customer service, and orientation to reward [16]. In the analyzed research, the following characteristics of the supportive organizational culture promoting knowledge sharing stood out.

Despite the fact that organizational culture and the origin of organizational climate concepts are different methodological traditions, in the management paradigm several theoretical and empirical tests to combine the two concepts can be found: both organizational culture and climate reveal the macroperspective of the organizational context where shared experiences develop that affect the end result sought by the organization [79] If an organization does not create a context suitable for knowledge sharing, knowledge management activities will simply fail [50].The analyzed scientific works highlight the separation of organizational culture and climate, as a context that promotes knowledge sharing, through the dimension of individual perception. Organizational climate is the result of the interaction of individuals and their environment, shaping individual behavior and influencing intentions to share or not to share knowledge [78,80]. and it is therefore important to understand that the climate perceived by organizational members can vary radically [81,82]. Thus, it can be stated that in the perspective of knowledge sharing, the authors follow the approach formed by Zohar and Hofmann (2012) when the organizational climate is treated as the perception of individuals’ organizational values and priorities, i.e., the culture of the organization reflects what the organization believes in, and the climate of the organization expresses how individual employees perceive the culture of the organization.

Research reveals that organizations with a culture of team-oriented work are more successful in sharing knowledge than those that are more focused on fully technology-based decision-making [74]. This insight confirms that the externalization of knowledge from tacit to explicit knowledge takes place in close (physical) interaction of the members of the organization, engaging in knowledge sharing to achieve common goals. An integral part of the social sharing process is the shared language between the members of the organization, expressing the various subtleties, acronyms, and hidden meanings, formed by the members of the organization acting in the same context and enabling members to acquire, convert, and apply new knowledge in the context in which it has a specific meaning [74].

External motivation of members of the organization as a supportive characteristic of knowledge sharing encourages members of the organization to participate in knowledge management activities and stimulates not only the sharing of goals, visions, and tasks, but also knowledge-sharing activities [17].

A culture of knowledge sharing is characterized by a high level of trust among members of an organization [19]. Interpersonal trust between members of an organization is the basis of their relationship, creating an opportunity for socialization [83] and a positive effect on knowledge sharing, as members of an organization tend to share knowledge only with those they trust [17,51]. A higher level of trust creates stronger relationships not only between members of the organization but also between members of the organization and the organization [18]. The positive relationship of the members of the organization with the organization is expressed by their commitment to the organization, which in the organizational culture perspective is defined as the emotional attachment of the organization members to the organization and identification with the organization’s values, which leads to a member feeling responsibility to help the organization to achieve its goals [18] empowering their self-efficacy and dedicating themselves to the task [84].

Transformational leadership: The role of a leader in an organization is one of the keys to creating the preconditions for knowledge management activities through management support, focused on inspiring employees to share knowledge and support actions that ensure such activities [51,83]. Qualitative analysis of the research revealed that knowledge management activities in an organization are ensured by a transformational leadership style characterized by motivating members of the organization for autonomy, mutual trust and “cultivation” of the commitment to the organization [50,85]. Transformational leaders create a supportive environment and, through charisma and special attention to employees, promote their intellectual development, motivate the creation and sharing of knowledge, develop a learning culture and discipline, and create mechanisms for knowledge management activities [17,80,84,86].Some authors identify transformational leadership styles with knowledge-oriented leadership, thus emphasizing the importance of this leadership style for ensuring knowledge management activities in an organization [86,87].

Less formalized and centralized organizational structure. The organizational structure is “responsible” for formalizing the explicit knowledge, the level of autonomy of the members of the organization, uniting them by specific means, desertification, and selection of effective communication channels for knowledge flows [49]. According Arif et al. (2015), organizational structures are most often categorized by formality, centrality, and integration [17]. Effective knowledge sharing requires a more flexible organizational structure, diversified teams of organization’s members, and common goals that link them [19]. When the organizational structure is less formalized and centralized and more integrated, a higher level of social interaction between members of the organization is achieved and the conductivity of knowledge sharing between functionally and hierarchically different members of the organization is ensured [83].

#### 3.3.3. Individual Enablers

Knowledge sharing is not possible in any organization without interacting individuals pursuing a goal that unites them. In particular, in the absence of individuals with tacit knowledge who at the individual level do not show knowledge-sharing behavior, an organization’s knowledge management skills are not possible. Qualitative analysis of research papers revealed that the following personal characteristics are important for individual behavior of knowledge sharing: openness to experience [78], satisfaction with helping others [26,51].; self-efficacy [26]; attitudes, subjective norms, and conscious behavior control [51].; internal motivation to share knowledge [26,51,88,89].

### 3.4. Integration

**Figure 2:**
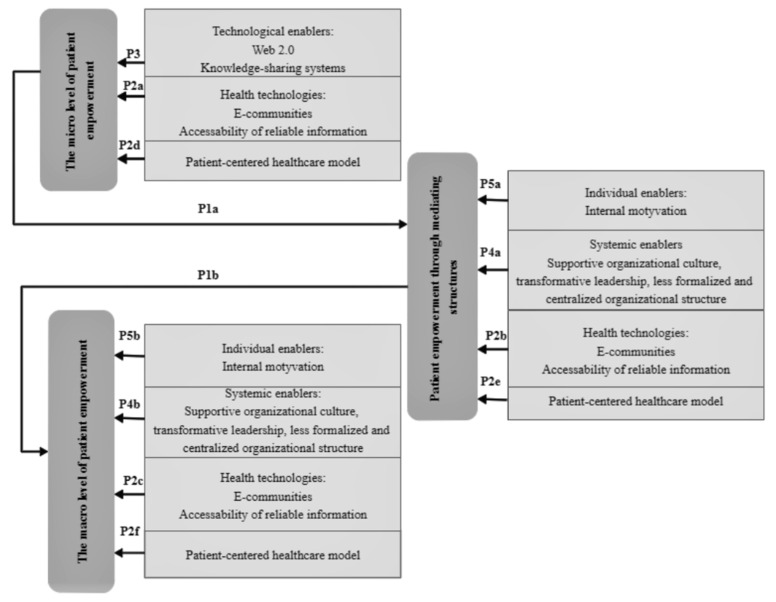
Enablers of patient knowledge empowerment for self-management of chronic disease framework. Note: P1a and P1b relationships are grounded by Proposition 1; P2a, P2b, P2c, P2d, P2e, and P2f relationships are grounded by Proposition 2; P3 relationship is grounded by Proposition 3; P4a and P4b relationships grounded by Proposition 4; and P5a and P5b relationships grounded by Proposition 5.

During the integrative review, having isolated patient knowledge enablers from public health management and knowledge management research papers, they were integrated through patient empowerment levels. Based on the relationships between patient empowerment levels and patient knowledge enablers identified during the integrative literature review, propositions were formulated and substantiated, illustrating them by Enablers of Patient Knowledge Empowerment for Self-Management of Chronic Disease Framework (see Figure 2).

**Proposition** **1.** *Patient empowerment through mediating structures mediates the relation between the patient empowerment micro level (P1a) and the empowerment macro level (P1b).*

Qualitative analysis of the research revealed that patient empowerment evolves through three levels of empowerment, in which the patient becomes increasingly empowered to use their knowledge in everyday decisions to pursue not only their own but also community health behaviors [14,18,51,60,74,90,91].

Patient progress across empowerment levels can be equated with the trajectory that defines the patient empowerment process when a patient from a passive recipient of information becomes an active healthcare partner. First of all, the microlevel of empowerment is formed, which includes the patient’s psychological empowerment, manifested in the patient’s perception of themself as being able to consciously influence their behavior in a health-friendly direction. Patient’s self-efficacy has a positive effect on their confidence to objectively assess their knowledge and cognitive abilities in the areas of health literacy. On this basis, the interactive element of patient empowerment is beginning to emerge, with the aim of transferring existing knowledge and cognitive abilities to a community of patients with the same chronic disease through mediating structures such, for example, as informal patient organizations/groups. Active participation of patients in different groups in sharing their knowledge and developing critical awareness of chronic illness and health, according to Menon and George (2018), is crucial in harnessing patient knowledge and thus shaping patient satisfaction so that they are empowered to take daily decisions related to their illness [59].

According to Rissel (1996) provisions on continuum empowerment, it can be argued that having empowered patients through mediating structures, patient empowerment evolves from the field of psychological empowerment to the field of community empowerment when mediating structures become community organizations embracing the expertise of empowered patients on the basis of which social conditions are created and changed [36].

**Proposition** **2.** *Health technologies (P2a, P2b, and P2c) and the patient-centered healthcare model (P2d, P2e, and P2f) act as enablers of patient knowledge across all levels of patient empowerment.*

As can be seen in Figure 2, qualitative analysis of the research revealed a link between patient knowledge enablers such as preconditions created by health technology (e-communities and accessibility of reliable information) [16,51,59,60,68,74] the patient-centered healthcare model [14,92] and all levels of patient empowerment.

Health technologies create the preconditions for e-communities to appear, for patients to access reliable information related to their chronic disease and create an effective environment for face-to-face interaction. The accessibility of reliable information increases patients’ self-efficacy and self-confidence in decision-making, and e-communities ensure knowledge sharing through patient interaction with healthcare professionals and other patients. At all levels of patient empowerment, the availability of reliable information about a chronic disease is very important to the patient, because whether they are in the field of psychological empowerment or already in the community empowerment, they face their chronic disease on a daily basis and have to make health decisions on the basis of reliable, related, and relevant information. Meanwhile, interaction through e-communities varies depending on the level of empowerment the patient is at. At the microlevel of patient empowerment, patient knowledge empowerment is dominated through interaction with sharing knowledge with the healthcare professional and passive monitoring of the activities of virtual patient communities. At other levels of patient empowerment, knowledge is empowered through active interaction not only with the healthcare professional, but also the transfer of existing knowledge to members of (informal) virtual communities and community organizations.

Given that the goal of the patient-centered healthcare model is to involve the patient in the treatment and day-to-day management of the chronic disease in the most acceptable way, qualitative analysis of the research revealed that this patient knowledge enabler operates across all levels of patient empowerment because as patient empowerment evolves, patient engagement unequivocally increases.

**Proposition** **3.** *Patient knowledge empowerment at the microempowerment level is stimulated by technological knowledge sharing enablers (P3).*

The analysis of selected research papers revealed that there is a link between technological knowledge-sharing enablers such as Web 2.0 and the knowledge-sharing systems and the microlevel of patient empowerment [65].The latest finding of the study suggests that at the microlevel of patient empowerment, the above-mentioned technological knowledge enablers, acting on the basis of already discussed health technologies, stimulate codification of patient and healthcare professional knowledge in order for the patient to express accumulated knowledge, experience in everyday life struggling with chronic disease and objective information on health parameters; and the healthcare professional, after assessing the knowledge expressed by the patient, could convey recommendations based on knowledge and experience for making daily decisions in the most understandable and acceptable way for the patient.

**Proposition** **4.** *Patient knowledge empowerment through mediating structures (P4a) and at the macrolevel (P4b) is stimulated by systemic knowledge sharing enablers.*

The study revealed that to stimulate knowledge sharing in mediating structures and at macro level for patient knowledge empowerment transformational leadership manifestations are important [17,51,84,93], supportive culture of these organizations [16,18,51,74,91,93], and a less formalized and centralized structure of the organizations [17,74,93]. Regardless of the fact that mediating patient structures are mostly informal and patient community organizations are formalized, through a less formalized and centralized organizational structure, active social interaction between members of the organization is created to ensure knowledge sharing. The supportive organizational culture and the manifestation of transformational leadership in the organization are aimed at changing the behavior of the members of the organization, stimulating patients’ orientation to continuous learning, and sharing knowledge, skills, and competencies in the context of learning health system.

**Proposition** **5.** *Patient knowledge empowerment through mediating structures (P5a) and at the macrolevel (P5b) is stimulated by the individual knowledge sharing enabler–internal patient motivation.*

An integrated analysis of research reveals that knowledge sharing enabled by patients through mediating structures and at the macrolevel is stimulated by their internal motivation [51]. In other words, as patient empowerment evolves across the levels discussed above, patient‘s awareness of the importance of knowledge sharing in the interpersonal dimension is built, where an internal stimulus is felt to transfer existing knowledge and cognitive skills to the same chronic disease community and later to address related social issues.

### 3.5. Limitations

The limitations of the performed integrative review, which could potentially influence the results of the research, can be related to the following methodological choices:

- Given the time and physical resources of the researchers, a relatively limited number of databases for the identification of potentially eligible studies were used.
- The choice to analyze only open access publications due to limited financial resources may have led to insufficient identification of themes of knowledge enablers for chronic patients from public health management and knowledge management perspectives and thus may have affected the results of the integration of these two perspectives.

## 4. Conclusions

Patient empowerment takes place across the levels of empowerment, and the result of patient empowerment primarily is the psychological empowerment of individuals and then that of community, creating organizations involving stakeholders, which operate through interactions to achieve health-related goals. Patients’ knowledge is formed in a process that includes the search for knowledge about their disease, its synthesis, and the use of specific knowledge. Knowledge empowerment also takes place in a process in which the patient, across the levels of empowerment, acquires the power to purposefully use their knowledge in everyday decisions to achieve health behavior.

Enablers of patient knowledge empowerment for self-management of chronic disease framework confirms the importance of looking at patient knowledge empowerment as a process, as patient empowerment evolves consistently across patient empowerment levels. The above-mentioned model also reveals that at all levels of patient empowerment there are both knowledge enablers distinguished in public health management theory and knowledge enablers distinguished in knowledge management theory. Thus, in the context of patient empowerment, it is important to look at patient knowledge empowerment from both a patient perspective and an organizational perspective when searching for solutions to stimulate the use of knowledge, as the integration of these two perspectives provides a holistic frame for patient knowledge empowerment.

## Figures and Tables

**Table 1:** Search sources and results.

Databases	Number of Articles Found	Article Type	Subject Areas	Access Type, Review Type
**ScienceDirect**	51	Review articles (14) Research articles (34) Discussion (2) Editorials (1)	Medicine and Dentistry (29) Computer Science (6) Nursing and Health Professionals (9) Biochemistry, Genetics and Molecular Biology (3) Immunology and Microbiology (2) Neuroscience (2)	Open Access, Peer-review
**Emerald Management**	56	Articles (56)	Social Sciences (56)	Open Access, Peer-review

**Table 2:** Synthesis of identified themes in public health management literature (source: own elaboration).

Themes	Subthemes	Number of Citations
Individual enablers	Autonomy	8
	Motivation	3
	Involvement in the search for a health-related decision	4
	Involvement of a partner	3
	Self-esteem	2
	Clear available information about the chronic disease	2
Health technologies	Digital information	11
	Digital self-care via empowering healthcare forms	36
	Accessibility of reliable information	11
	Creation of e-communities	10
Patient-centeredness model	Characteristics of patient-centered model	12
Disease management programs	Multicomponentiality of disease management programs	8
	Interaction between the patient and a healthcare professional	4
Learning health system	Mission of learning health system	9
	World Health Organization	5

**Table 3:** Synthesis of identified themes in knowledge management literature (source: own elaboration).

Themes	Subthemes	Number of Citations
Technological enablers of knowledge sharing	Web 2.0	12
	Systems of knowledge sharing	5
Systemic enablers of knowledge sharing	Supportive organizational culture	38
	Transformative leadership	15
	Less formalized and centralized organizational structure	7
Enablers of individual knowledge sharing	Openness to experience	3
	Satisfaction arising from helping others	3
	Self-efficacy	1
	Attitudes, subjective norms and conscious self-control	2
	Internal motivation for knowledge sharing	7

**Table 4:** Synthesis of topics for patient empowerment levels (source: own elaboration).

Theme	Subthemes	Number of Citations:	
		In Public Health Management Literature	In Knowledge Management Literature
**Levels of patient empowerment**	Microlevel	11	6
	Empowerment through mediating structures	15	23
	Macrolevel	9	19

## Data Availability

The study materials and the detail of all analyses are available from the corresponding author upon reasonable request.

## References

[1] Holmström I., Röing M. (2010). The relation between patient-centeredness and patient empowerment: A discussion on concepts. Patient Educ. Couns..

[2] Small N., Bower P., Chew-Graham C.A., Whalley D., Protheroe J. (2013). Patient empowerment in long-term conditions: Development and preliminary testing of a new measure. BMC Health Serv. Res..

[3] Schulz P.J., Nakamoto K. (2013). Patient behavior and the benefits of artificial intelligence: The perils of “dangerous” literacy and illusory patient empowerment. Patient Educ. Couns..

[4] Anderson R.M., Funnell M.M. (2010). Patient empowerment: Myths and misconceptions. Patient Educ. Couns..

[5] Asimakopoulou K., Gilbert D., Newton P., Scambler S. (2012). Back to basics: Re-examining the role of patient empowerment in diabetes. Patient Educ. Couns..

[6] Wakefield D., Bayly J., Selman L.E., Firth A.M., Higginson I.J., Murtagh F.E. (2018). Patient empowerment, what does it mean for adults in the advanced stages of a life-limiting illness: A systematic review using critical interpretive synthesis. Palliat. Med..

[7] Snyder H. (2019). Literature review as a research methodology: An overview and guidelines. J. Bus. Res..

[8] Köhler A.K., Tingström P., Jaarsma T., Nilsson S. (2018). Patient empowerment and general self-efficacy in patients with coronary heart disease: A cross-sectional study. BMC Fam. Pr..

[9] Martos-Méndez M.J. (2015). Self-efficacy and adherence to treatment: The mediating effects of social support. J. Behav. Health Soc. Issues.

[10] Aujoulat I., D’Hoore W., Deccache A. (2007). Patient empowerment in theory and practice: Polysemy or cacophony?. Patient Educ. Couns..

[11] Piper S. (2010). Patient empowerment: Emancipatory or technological practice?. Patient Educ. Couns..

[12] Johnston A.C., Worrell J.L., Di Gangi P.M., Wasko M. (2013). Online health communities. Inf. Technol. People.

[13] Ippolito A., Smaldone F., Ruberto M. (2019). Exploring patient empowerment. TQM J..

[14] Doherty K., Barry M., Belisario J.M., Morrison C., Car J., Doherty G. (2020). Personal information and public health: Design tensions in sharing and monitoring wellbeing in pregnancy. Int. J. Hum. Comput. Stud..

[15] Christie H.L., Martin J.L., Connor J., Tange H.J., Verhey F.R., De Vugt M.E., Orrell M. (2019). eHealth interventions to support caregivers of people with dementia may be proven effective, but are they implementation-ready?. Internet Interv..

[16] Adeinat I.M., Abdulfatah F.H. (2019). Organizational culture and knowledge management processes: Case study in a public university. VINE J. Infect. Knowl. Manag. Syst..

[17] Arif M., Mohammed A.-Z., Gupta A.D. (2015). Understanding knowledge sharing in the Jordanian construction industry. Constr. Innov..

[18] Curado C., Vieira S. (2019). Trust, knowledge sharing and organizational commitment in SMEs. Pers. Rev..

[19] Yao J., Crupi A., Di Minin A., Zhang X. (2020). Knowledge sharing and technological innovation capabilities of Chinese software SMEs. J. Knowl. Manag..

[20] Scharf D. (2010). A New View of Patient Education: How Information and Knowledge Management Can Contribute to Pa-tient-centered Health Care. Knowledge Work.

[21] Menear M., Blanchette M.-A., Demers-Payette O., Roy D. (2019). A framework for value-creating learning health systems. Health Res. Policy Syst..

[22] Friedman C.P., Wong A.K., Blumenthal D. (2010). Achieving a Nationwide Learning Health System. Sci. Transl. Med..

[23] Rubin J.C. (2017). Patient empowerment and the Learning Health System. Learn. Health Syst..

[24] Flynn A.J., Friedman C.P., Boisvert P., Landis-Lewis Z., Lagoze C. (2018). The Knowledge Object Reference Ontology (KORO): A formalism to support management and sharing of computable biomedical knowledge for learning health systems. Learn. Health Syst..

[25] Nonaka I., Von Krogh G. (2009). Perspective—Tacit Knowledge and Knowledge Conversion: Controversy and Advancement in Organizational Knowledge Creation Theory. Organ. Sci..

[26] Hussein A.T.T., Singh S.K., Farouk S., Sohal A.S. (2016). Knowledge sharing enablers, processes and firm innovation capability. J. WorkLearn..

[27] Kale S., Karaman E.A. (2011). Evaluating the Knowledge Management Practices of Construction Firms by Using Importance–Comparative Performance Analysis Maps. J. Constr. Eng. Manag..

[28] Gutiérrez L.M., Delois K.A., GlenMaye L. (1995). Understanding Empowerment Practice: Building on Practitioner-Based Knowledge. Fam. Soc. J. Contemp. Soc. Serv..

[29] Peterson N.A. (2014). Empowerment Theory: Clarifying the Nature of Higher-Order Multidimensional Constructs. Am. J. Community Psychol..

[30] European Patient Forum, Toolkit for Patient Organizations on Patient Empowerment. https://www.eu-patient.eu/library/toolkits/.

[31] Bate P., Robert G. (2006). Experience-based design: From redesigning the system around the patient to co-designing services with the patient. Qual. Saf. Health Care.

[32] Sharma S., Khadka A. (2019). Role of empowerment and sense of community on online social health support group. Infect. Technol. People.

[33] Rutten L.J.F., Hesse B.W., Sauver J.L.S., Wilson P., Chawla N., Hartigan D.B., Moser R.P., Taplin S., Glasgow R., Arora N.K. (2016). Health Self-Efficacy Among Populations with Multiple Chronic Conditions: The Value of Patient-Centered Communication. Adv. Ther..

[34] Wagstaff B. (2006). Impact of antibiotic restrictions: The patient’s perspective. Clin. Microbiol. Infect..

[35] Mora M.A., Saarijärvi M., Sparud-Lundin C., Moons P., Bratt E.-L. (2020). Empowering Young Persons with Congenital Heart Disease: Using Intervention Mapping to Develop a Transition Program—The Stepstones Project. J. Pediatr. Nurs..

[36] Rissel C. (1994). Empowerment: The holy grail of health promotion?. Health Promot. Int..

[37] Hatch M.J., Cunliffe A.L. (2013). Organization Theory: Modern, Symbolic, and Postmodern Perspectives.

[38] Dizon J.T. (2012). Theoretical Concepts and Practice of Community Organizing. J. Public Aff. Dev..

[39] Field B., Booth A., Ilott I., Gerrish K. (2014). Using the Knowledge to Action Framework in practice: A citation analysis and systematic review. Implement. Sci..

[40] Milat A.J., Li B. (2017). Narrative review of frameworks for translating research evidence into policy and practice. Public Health Res. Pr..

[41] Brathwaite R., Hutchinson E., McKee M., Palafox B., Balabanova D. (2020). The Long and Winding Road: A Systematic Literature Review Conceptualising Pathways for Hypertension Care and Control in Low- and Middle-Income Countries. Int. J. Health Policy Manag..

[42] Gold A.H., Malhotra A., Segars A.H. (2001). Knowledge Management: An Organizational Capabilities Perspective. J. Manag. Inf. Syst..

[43] Song J.H., Yoon S.W., Yoon H.J. (2011). Identifying organizational knowledge creation enablers through content analysis: The voice from the industry. Perform. Improv. Q..

[44] Lee H., Choi B. (2003). Journal of Management Information Systems, Informa-tion and Management. J. Organ. Comput. Electron. Commer..

[45] Al-Gharibeh K. (2011). The Knowledge Enablers of Knowledge Transfer: An Empirical Study in Telecommunications Companies. IBIMA Bus. Rev..

[46] Owusu-Manu D.-G., Edwards D.J., Pärn E.A., Antwi-Afari M.F., Aigbavboa C. (2018). The knowledge enablers of knowledge transfer: A study in the construction industries in Ghana. J. Eng. Des. Technol..

[47] Lilleoere A., Hansen E.H. (2011). Knowledge-sharing enablers and barriers in pharmaceutical research and development. J. Knowl. Manag..

[48] Karamat J., Shurong T., Ahmad N., Waheed A., Mahmood K. (2018). Enablers Supporting the Implementation of Knowledge Management in the Healthcare of Pakistan. Int. J. Environ. Res. Public Health.

[49] Gonzalez R.V.D., Melo T.M. (2017). Linkage between dynamics capability and knowledge management factors. Manag. Decis..

[50] Donate M.J., Guadamillas F. (2011). Organizational factors to support knowledge management and innovation. J. Knowl. Manag..

[51] Ali A.A., Selvam D.D.D.P., Paris L., Gunasekaran A. (2019). Key factors influencing knowledge sharing practices and its relationship with organizational performance within the oil and gas industry. J. Knowl. Manag..

[52] Cronin M.A., George E. (2020). The Why and How of the Integrative Review. Organ. Res. Methods.

[53] Lewins A., Silver C. (2014). Using Software in Qualitative Research: A Step-by-Step Guide.

[54] Seidel J.V. QDA: A Model of the Process Noticing, Collecting, Thinking about Things. http://eer.engin.umich.edu/wp-content/uploads/sites/443/2019/08/Seidel-Qualitative-Data-Analysis.pdf.

[55] Meslin E.M., Alpert S.A., Carroll A.E., Odell J.D., Tierney W.M., Schwartz P.H. (2013). Giving patients granular control of personal health information: Using an ethics ‘Points to Consider’ to inform informatics system designers. Int. J. Med. Inform..

[56] Meis J.J., Bosma C.B., Spruit M.A., Franssen F.M., Janssen D.J., Teixeira P.J., Augustin I.M., Wouters E.F., De Vries N.K., Schols A.M. (2014). A qualitative assessment of COPD patients’ experiences of pulmonary rehabilitation and guidance by healthcare professionals. Respir. Med..

[57] Steinmetz M., Rammos C., Rassaf T., Lortz J. (2020). Digital interventions in the treatment of cardiovascular risk factors and atherosclerotic vascular disease. IJC Hear. Vasc..

[58] Kraus S., Schiavone F., Pluzhnikova A., Invernizzi A.C. (2021). Digital transformation in healthcare: Analyzing the current state-of-research. J. Bus. Res..

[59] Menon M., George B. (2018). Social media use for patient empowerment in the Gulf Cooperation Council region. Clin. eHealth.

[60] Kouroubali A., Katehakis D.G. (2019). The new European interoperability framework as a facilitator of digital transformation for citizen empowerment. J. Biomed. Inform..

[61] Verbraecken J. (2016). Telemedicine Applications in Sleep Disordered Breathing. Sleep Med. Clin..

[62] Marco-Ruiz L., Bønes E., De La Asunción E., Gabarron E., Aviles-Solis J.C., Lee E., Traver V., Sato K., Bellika J.G. (2017). Combining multivariate statistics and the think-aloud protocol to assess Human-Computer Interaction barriers in symptom checkers. J. Biomed. Inform..

[63] Hatton J.D., Schmidt T.M., Jelen J. (2012). Adoption of Electronic Health Care Records: Physician Heuristics and Hesitancy. Procedia Technol..

[64] Van Gorp P., Comuzzi M., Jahnen A., Kaymak U., Middleton B. (2014). An open platform for personal health record apps with platform-level privacy protection. Comput. Biol. Med..

[65] Kopanitsa G., Veseli H., Yampolsky V. (2015). Development, implementation and evaluation of an information model for archetype based user responsive medical data visualization. J. Biomed. Inform..

[66] Genitsaridi I., Kondylakis H., Koumakis L., Marias K., Tsiknakis M. (2013). Towards Intelligent Personal Health Record Systems: Review, Criteria and Extensions. Procedia Comput. Sci..

[67] Fiske A., Buyx A., Prainsack B. (2020). The double-edged sword of digital self-care: Physician perspectives from Northern Germany. Soc. Sci. Med..

[68] Wicks P., Keininger D.L., Massagli M.P., De La Loge C., Brownstein C., Isojärvi J., Heywood J. (2012). Perceived benefits of sharing health data between people with epilepsy on an online platform. Epilepsy Behav..

[69] Van Der Heide I., Snoeijs S., Quattrini S., Struckmann V., Hujala A., Schellevis F., Rijken M. (2018). Patient-centeredness of integrated care programs for people with multimorbidity. Results from the European ICARE4EU project. Health Policy.

[70] Pimouguet C., Le Goff M., Thiébaut R., Dartiques J.F., Helmer C. (2011). Effectiveness of disease-management programs for improving diabetes care: A meta-analysis. CMAJ..

[71] CN B.M., Alberts M.J., Balady G.J., Ballantyne C.M., Berra K., Black H.R., Underberg J.A. (2009). ACCF/AHA/ACP 2009 Competence and Training Statement: A Curriculum on Prevention of Cardiovascular Disease. J. Am. Coll. Cardiol..

[72] Holt D., Bouder F., Elemuwa C., Gaedicke G., Khamesipour A., Kisler B., Kochhar S., Kutalek R., Maurer W., Obermeier P. (2016). The importance of the patient voice in vaccination and vaccine safety—are we listening?. Clin. Microbiol. Infect..

[73] Kruk M.E., Gage A.D., Arsenault C., Jordan K., Leslie H.H., Roder-DeWan S., Adeyi O., Barker P., Daelmans B., Doubova S.V. (2018). High-quality health systems in the Sustainable Development Goals era: Time for a revolution. Lancet Glob. Health.

[74] Al Saifi S.A. (2015). Positioning organisational culture in knowledge management research. J. Knowl. Manag..

[75] Malik M.S., Kanwal M. (2018). Impacts of organizational knowledge sharing practices on employees’ job satisfaction. J. Work. Learn..

[76] Qandah R., Suifan T.S., Masa’Deh R., Obeidat B.Y. (2020). The impact of knowledge management capabilities on innovation in entrepreneurial companies in Jordan. Int. J. Organ. Anal..

[77] Yeh Y., Lai S., Ho C. (2006). Knowledge management enablers: A case study. Ind. Manag. Data Syst..

[78] Han S.-H. (2018). The antecedents and dimensionality of knowledge-sharing intention. Eur. J. Train. Dev..

[79] Ehrhart M.G., Schneider B. (2013). Organizational Climate and Culture. Annu. Rev. Psychol..

[80] Pellegrini M.M., Ciampi F., Marzi G., Orlando B. (2020). The relationship between knowledge management and leadership: Mapping the field and providing future research avenues. J. Knowl. Manag..

[81] De Angelis C.T. (2016). The impact of national culture and knowledge management on governmental intelligence. J. Model. Manag..

[82] Mutonyi B.R., Slåtten T., Lien G. (2020). Organizational climate and creative performance in the public sector. Eur. Bus. Rev..

[83] Joshi H., Chawla D., Farooquie J.A. (2014). Segmenting knowledge management (KM) practitioners and its relationship to performance variation-some empirical evidence. J. Knowl. Manag..

[84] Gürlek M., Çemberci M. (2020). Understanding the relationships among knowledge-oriented leadership, knowledge management capacity, innovation performance and organizational performance. Kybernetes.

[85] Xiao Y., Zhang X., De Pablos P.O. (2017). How does individuals’ exchange orientation moderate the relationship between transformational leadership and knowledge sharing?. J. Knowl. Manag..

[86] Latif K.F., Afzal O., Saqib A., Sahibzada U.F., Alam W. (2020). Direct and configurational paths of knowledge-oriented leadership, entrepreneurial orientation, and knowledge management processes to project success. J. Intellect. Cap..

[87] Rohim A., Budhiasa I.G.S. (2019). Organizational culture as moderator in the relationship between organizational reward on knowledge sharing and employee performance. J. Manag. Dev..

[88] Wong K.Y., Aspinwall E. (2005). An empirical study of the important factors for knowledge-management adoption in the SME sector. J. Knowl. Manag..

[89] Thani F.N., Mirkamali S.M. (2018). Factors that enable knowledge creation in higher education: A structural model. Data Technol. Appl..

[90] Ramachandran S.D., Chong S., Wong K. (2013). Knowledge management practices and enablers in public universities: A gap analysis. Campus-Wide Infect. Syst..

[91] Huang Y.-C., Chin Y.-C. (2018). Transforming collective knowledge into team intelligence: The role of collective teaching. J. Knowl. Manag..

[92] Paduch A., Kuske S., Schiereck T., Droste S., Loerbroks A., Sørensen M., Maggini M., Icks A. (2017). Psychosocial barriers to healthcare use among individuals with diabetes mellitus: A systematic review. Prim. Care Diabetes.

[93] Akbari N., Ghaffari A. (2017). Verifying relationship of knowledge management initiatives and the empowerment of human resources. J. Knowl. Manag..

